# Ultrafast Thermal Nonlinearity

**DOI:** 10.1038/srep17899

**Published:** 2015-12-08

**Authors:** Jacob B. Khurgin, Greg Sun, Wei Ting Chen, Wei-Yi Tsai, Din Ping Tsai

**Affiliations:** 1 Department of Electrical and Computer Engineering, Johns Hopkins University, Baltimore, Maryland 21218; 2 Department of Engineering, University of Massachusetts Boston, Boston, Massachusetts 02125; 3 Department of Physics, National Taiwan University, Taipei 10617, Taiwan; 4 Research Center for Applied Sciences, Academia Sinica, Taipei 11529, Taiwan

## Abstract

Third order nonlinear optical phenomena explored in the last half century have been predicted to find wide range of applications in many walks of life, such as all-optical switching, routing, and others, yet this promise has not been fulfilled primarily because the strength of nonlinear effects is too low when they are to occur on the picosecond scale required in today’s signal processing applications. The strongest of the third-order nonlinearities, engendered by thermal effects, is considered to be too slow for the above applications. In this work we show that when optical fields are concentrated into the volumes on the scale of few tens of nanometers, the speed of the thermo-optical effects approaches picosecond scale. Such a sub-diffraction limit concentration of field can be accomplished with the use of plasmonic effects in metal nanoparticles impregnating the thermo-optic dielectric (e.g. amorphous Si) and leads to phase shifts sufficient for all optical switching on ultrafast scale.

Intelligent exploitation of nonlinear optical phenomena^1^ provides a theoretical basis for achieving ultra-fast all-optical control in optoelectronic devices, enabling novel system architectures for signal processing and communications. The most common nonlinear optical effect is the change of refractive index *n* caused by the irradiance (power density) of optical wave I as Δ*n* = *n*_2_*I* where *n*_2_ is the nonlinear refractive index. The nonlinear index change is a third-order nonlinear optical effect (often called the Optical Kerr effect)^2^ in which the optical polarization ***P*** is proportional to the cube of the optical field strength ***E*** as 
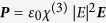
 where *χ*^(3)^ is the third order susceptibility.

A strong optical field will change both real and imaginary parts of the refractive index . If the optical field is monochromatic then the most relevant phenomena are self-phase-modulation SPM (a change in real part of index) and nonlinear absorption (a change in imaginary part of index), both of which can be used in optical thresholding and limiting. A more versatile effect, cross-phase modulation (XPM) occurs when two optical waves of different frequencies (or polarizations) propagate in the nonlinear material, opening possibility for all-optical switching, as well as signal regeneration. In addition, if the change of refractive index, Δ*n* can be made to occur quickly enough to follow the beating of two optical signals at frequency *ω*_1_ − *ω*_2_, yet another manifestation of *χ*^(3)^– four wave mixing (FWM) – becomes possible: the optical power gets coherently coupled into the new waves at frequencies, 2*ω*_1_ − *ω*_2_ and 2*ω*_2_ − *ω*_1_, with obvious applications in all-optical frequency conversion.

Considerable effort has been expended on development of nonlinear optical materials whose response is simultaneously strong and fast, yet to this date the promise of all-optical devices remains largely unfulfilled. It is easy to see that any practical nonlinear optical devices (including all mentioned above) can operate efficiently only when the nonlinear phase shift ΔΦ = Δ*nωL*/*c* where *c* is the speed of light and *L* is the propagation length, can be of the order of *π*/2. The propagation length, however, is limited by the absorption; materials with higher nonlinear susceptibilities are characterized by the large absorption. As a result, the most successful fast third-order nonlinear medium today is the optical fiber^3^,^4^ where the relatively low nonlinear index is more than compensated for by the extraordinarily long propagation length, which, of course, makes the devices too large to integrate in a practical optical circuit. The second most promising approach is the semiconductor optical amplifier (SOA), in which low absorption is simply compensated by gain. But the SOA is impaired by high power dissipation, excessive noise, and speed that is lower than other nonlinear materials. Although significant strides have been made with waveguides made from silicon^5^ or chalcogenide glass^6^, the length required to achieve the shift is still much too large, on the order of a centimeter or more; moreover, these waveguides suffer from two photon absorption.

From a practical perspective, in generating large optical fields required for nonlinear optical processes, one always tries to maximize the instantaneous power by using short pulses from a (typically mode-locked) laser of limited average power, with relatively small duty cycle. Since the average power remains low, this approach does not cause deleterious thermal effects. In principle, similar concentration of power can be performed in spatial, rather than in temporal domains, exploiting the field effect concentration on the local scale. One of the more promising ways of achieving this concentration is to utilize the Surface Plasmon Polariton (SPP) resonance with sub-wavelength metal nanoparticles and their combinations^7^,^8^. The SPP in metal nanoparticles is characterized by a resonance frequency *ω*_0_ that can be adjusted by choosing a properly engineered shape of the particle; and the linewidth (or damping) constant *γ* of the order of 10^14^ s^−1^ for noble metals. One can think of SPPs as giant polarizable atoms with resonant frequency *ω*_0_ and Q-factor *Q* = *ω*_0_/*γ* < 30. This model implies that when the frequency is near the resonance, the local field in the volumes immediately adjacent to the metal nanoparticles get enhanced by a factor of the order of *Q* relative to the space-averaged field. It follows then that the third-order nonlinear optical phenomena get enhanced by a factor of roughly *fQ*^4^, where *f* is the volume fraction occupied by the metal particles^9^. Additional enhancement can be attained using more sophisticated plasmonic structure, such as dimers^10^,^11^ consisting of a bigger nanoparticle (serving as a “nanoantenna”) coupled to another smaller nanoparticle (serving as “nanocavity”), in which the power gets further concentrated^12^. Effective nonlinearity in such structures gets enhanced by as much as *fQ*^6^. However, more careful analysis shows, that since this enhancement is achieved primarily due to the local power concentration, it works well only at low powers, while at higher powers the local energy concentration exceeds the optical damage threshold^13^. In fact, if one introduces the maximum attainable nonlinear index change in a given material as Δn_max_, the maximum attainable phase shift is just ΔΦ_max_ ~ *Q*Δ*n*_max_/*n*. Hence, attaining the “holy grail” of nonlinear optics of a 90 degree phase shift requires a normalized index change Δ*n*/*n* of 10% – clearly unfeasible in most conventional nonlinear materials, due either to optical breakdown or to saturation of nonlinearity^14^ as higher order susceptibilities (*χ*^(5)^, *χ*^(7)^ and so on) come into play. For ultra-fast optical nonlinearities, the nonlinear index change is typically much less 1%.

The reason for such a small change in refractive index is simply the fact that fast nonlinearity has what is called an “electronic” nature, i.e., the change of refractive index occurs due to a minute displacement of electronic cloud confined by the binding potential (which can be that of an atom or a crystal bond) while the potential itself stays unaffected. If, on the other hand, the binding potential itself changes, as indeed happens when the temperature increases and the distance between neighboring atom changes, the changes of index can be much larger, on the order of a few percent. For example, in silicon the thermo-optical coefficient ∂*n*/∂*T* is on the order of 1.4 × 10^−4^ *K*^−1^, meaning that a few percent change in refractive index is a clear possibility^15^, as witnessed by numerous works on thermal nonlinearity in various media^16^,^17^. A high coefficient can be attained in amorphous Si. Since thermal nonlinearity is notoriously slow^18^, it is often dismissed as only a potential enabling mechanism for ultra-fast all-optical switching. However, the intrinsic speed of thermal nonlinearity, i.e. the speed of relative motion of ions, is far from being low. The relative ion motion occurs on the scale of the optical phonon frequency, as fast as few hundred femtoseconds. What makes thermal nonlinearity slow is the rate of heat dissipation, typically on the microsecond to millisecond timescale. But this is only true when the heated region is relatively large, on the micrometer scale. With the use of SPPs one can concentrate the optical power and hence, the temperature, to the nanometer range, the rate of the dissipation can be reduced by many orders of magnitude, and the large index change Δ*n* can be induced thermally and quenched within a few picoseconds. That all kinds of diffusive processes, – e.g., thermal diffusion^19^ or carrier diffusion^20^ –occur on a faster time scale when the excitation is localized, is well known, but effects due to localization on a subwavelength scale have not been studied previously. We now introduce this new concept of ultrafast plasmon-assisted thermal nonlinearity.

## Plasmonic enhancement of thermal nonlinearity

Prior to getting into detailed description of thermal nonlinearity, we note that when the time scale of processes becomes shorter than the phonon scattering time, or when the length scale becomes comparable to the mean free path of phonons thermal transport, the phenomenon becomes ballistic rather than diffusive. Ballistic phonons do cause the time-averaged change of the refractive index, but they also scatter the light due to acousto-optic effect thus complicating the picture. Hence in this work we shall limit ourselves to the diffusive transport by choosing amorphous Si (a-Si) in which high frequency phonon scattering occurs on a picosecond scale^21^. Amorphous Si combines decent thermal conductivity with a large thermo-optic coefficient and thus seem to be suitable for our purposes. Ballistic regime^22^ requires separate consideration and we are planning to address it in future work.

**Figure 1:**
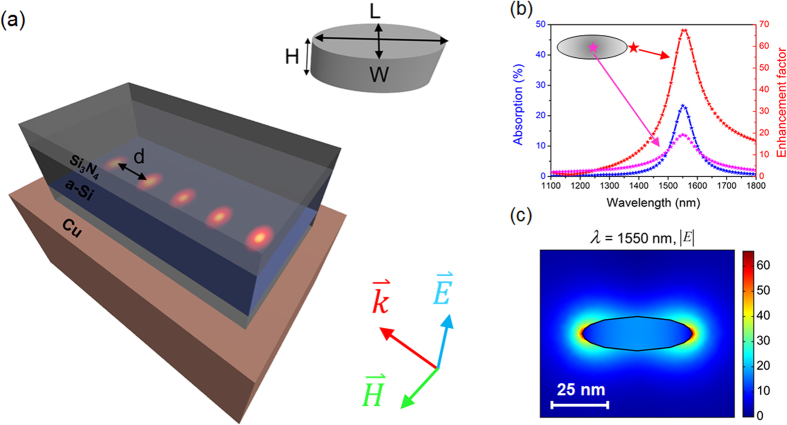
Absorption spectrum and field distribution in a-Si waveguide impregnated with Ag nanodisks. (**a**) Schematic diagram of Ag nanodisks embedded in a-Si waveguide with a cross section of 220 × 400 nm^2^ sandwiched between two Si_3_N_4_ layers of equal thickness of 165 nm. The waveguide sits on top of a copper heat sink. The nanodisks are separated from each other with a center-to-center *d* = 150 nm. Inset: Ag nanodisk with *L* = 48 nm, *W* = 15 nm, and *H* = 15 nm. (**b**) Optical absorption spectrum of a single Ag nanodisk in a-Si and average electric field enhancement inside of the Ag nanodisk. (**c**) Top view of the electric field distribution at *λ* = 1550 nm around Ag nanodisk (white scale bar dimension = 20 nm).

As an example, we consider the plasmonically enhanced structure shown in Fig. 1a. an amorphous silicon optical waveguide impregnated with *N*_np_ = 5 Ag nanoparticles, which are separated by a distance *d* = 150 nm from each other. The waveguide is placed between two Si_3_N_4_ cladding layers and rests on top of a copper heat sink. All nanoparticles have an elliptical disk shape with long axis (*L*), short axis (*W*) axes and thickness (*H*) of 48, 15, and 15 nm, respectively. When placed in a-Si the nanoparticles produce an SPP resonance at a wavelength of approximately 1550 nm for a propagating wave polarized along the long axis, as shown in the calculated absorption spectrum of Fig. 1b. The electric field distribution of Fig. 1c reveals field enhancement near the nanoparticle. As plotted in Fig. 1b, the field is enhanced resonantly with the full width at half maximum of about 100 nanometers, implying that *Q* ~ 16. The field enhancement inside the nanoparticle near its center has *Q*_met_ ~ 19, i.e. commensurate with *Q*, while the maximum enhancement occurs just outside of the nanoparticle pole where *Q*_max_ ~ 67.

To assure rapid cooling the waveguide is placed in thermal contact with a copper heat sink, with only a thin layer (165 nm) of Si_3_N_4_ separating the a-Si from the heat sink. Hence, the temperature rise in the bulk of waveguide averaged over the time is much less than the instantaneous rise near the nanoparticles, as our numerical modeling will show.

However, before embarking on exact numerical analysis, we wanted to obtain an order of magnitude estimate of the levels that might be reached using this thermo-optic effect. We first derived analytical expressions using spherical particles with equivalent volume, i.e. diameter 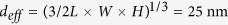 as a model. In the presence of metal nanoparticles, the effective dielectric constant of the waveguide material^12^, i.e. the ratio of volume-averaged electric displacement to volume-averaged electric field becomes


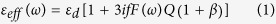


where 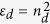 is the dielectric constant of α-Si, *f* is the fraction of the volume occupied by the metal nanoparticles, *Q* = *ω*_0_/*γ*, 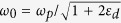 is the localized SPP resonance frequency, *ω*_*p*_ is the plasma frequency, *γ* is the scattering rate in the metal, 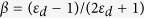 is a factor varying between 0 and 0.5 (*β* = 0.3 for Si), and the almost-Lorentzian (see Fig. 1b) spectral function is


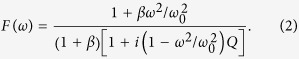


The analysis for elliptical particles will have different expressions for *ω*_0_ and *β* and their values will change, but it the order-of-magnitude results will not be affected. The fields both inside and outside the nanoparticle are resonantly enhanced by a factor proportional to 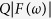 , a result confirmed by the numerical calculations shown in Fig. 1b. Thus, the electromagnetic energy in each nanoparticle is lost at a rate


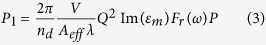


where *V* is the volume of one nanoparticle, *A*_*eff*_ is the effective cross-section of the waveguide, *P* is the average optical power in the waveguide, and 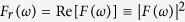 . The absorption coefficient per one nanoparticle is then


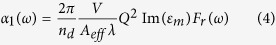


and the effective absorption coefficient per unit length is


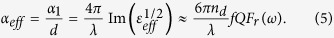


The absorption of the field inside the metal^23^,^24^ will cause the local temperature rise, which in turn will cause both the local change of the dielectric (a-Si) refractive index, Δ*n*_*d*_, and the change of the metal dielectric constant; this latter change occurs mostly because of the increase in the scattering rate Δ*γ*, augmented by small change in the background dielectric constant caused by off-resonant inter-band transitions. Both of these changes can cause modification of the effective dielectric constant, but the magnitudes of these modification differ significantly.

The change of scattering rate Δ*γ* causes broadening of the spectral shape *F*(*ω*) and hence a relatively small change in *Q*, according to Eq. (1), resulting in change of effective dielectric constant


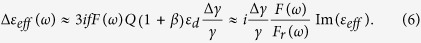


The all-optical switching in a few absorption lengths can occur only when 
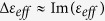
 which, according to Eq. (6), requires just about 100% relative change in the scattering rate in the metal, that cannot be realized in practice.

On the other hand, when the refractive index changes (Δ*n*_*d*_), it causes a change in the resonance frequency, and thus, a spectral shift in *F*(*ω*). The change in effective refractive index


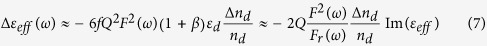


acquires additional resonant enhancement by a factor on the order of *Q*, when compared to Eq. (6). Therefore, all-optical switching within a few absorption lengths becomes feasible if the local relative index can be changed by a factor of roughly 1/2*Q* , i.e. only a few percent. Indeed, from Eqs (5) and (7) one can obtain the nonlinear phase shift for one absorption length as


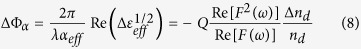


and the nonlinear change of absorption for one absorption length,


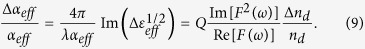


The maximum value for the phase shift ΔΦ_*α*_ = −*Q*Δ*n*_*d*_/*n*_*d*_ is obtained at the SPP resonance, while the maximum relative change of absorption, 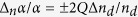, is obtained at a position one linewidth away from the resonance. Both are resonantly enhanced by a factor of *Q*; if we choose a representative value of *Q*, say 16, the resulting relative index change of 10% will be sufficient for optical switching. This large difference between exploiting the change in broadening and change in index stems from the nearly-Lorentzian line shape *F*(*ω*) in Fig. 1b, which is affected far more dramatically by the shift in resonance frequency than by the change in broadening. The background dielectric constant of the metal is engendered by the inter-band transition occurring in the UV band, far from the resonance. Hence the thermally induced change in this background dielectric constant is much smaller than the change of the dielectric constant of the surrounding dielectric. Furthermore, as evident from Fig. 1c, the field is concentrated outside the metal. Hence the shift of resonant frequency *ω*_0_ depends mostly on the change of the dielectric constant of the dielectric. Using the nonlinearity of the dielectric itself does have significant advantages, as we explain below.

## Analytical estimate of nonlinear phase shift and effective nonlinear index

We can now estimate the maximum phase shift that can be attained in one absorption length of the Ag nanoparticle impregnated waveguide of Fig. 1. First, we estimate the thermal diffusion time. The SPP mode “senses” the change of refractive index a region very close to the Ag nanoparticle surface. We assume that the effective size of the “active” region is twice the effective diameter of the nanoparticle, which leads to a heat diffusion time, 
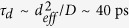
, where *D* = 0.1 cm^2^/*s* is the α-Si heat diffusivity. One can therefore choose the optical pulse width of *τ*_*p*_ ~ 20 ps in order to operate a tbit rate of 50 Gbit/s with a 50% duty cycle. The maximum temperature rise that can be tolerated in silver is about 1100 K (about 150 K below melting point), i.e. Δ*T* ~ 800 K from which it follows that Δ*n*_*d*_ ~ 0.2 and Δ*n*/*n*_*d*_ ~ 0.06. The phase shift over one absorption length is calculated as 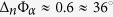. While this may not be quite sufficient for full optical switching from a 0-state (0% transmission) to a 1-state (100% transmission), a phase shift value = 0.6 –is sufficient to allow switching from 0% to 20% transmission.

Next we estimate the power in the waveguide required to achieve this minimum phase shift value for successful switching. Over the duration of the pulse the energy dissipated in the nanoparticle spreads into a layer around the surface of the nanoparticle, penetrating into the a- Si material. The thickness of this layer is comparable to the radius of the nanoparticle. Using specific heat per unit of volume in Si *c*_Si_ = 1.6 J/cm^3^ · K and Ag *c*_Ag_ = 2.1 J/cm^3^ · K the required energy dissipation per nanoparticle becomes 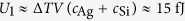. Therefore, the required power dissipation in one nanoparticle is 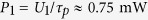, so the power that must be launched into the waveguide is only 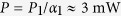, corresponding to peak intensity of less than 10 MW/cm^2^. The switching energy per pulse (or bit) is then 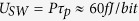.

The effective nonlinear index can be found as a ratio of the change in refractive index, 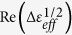 to the peak intensity in the waveguide,


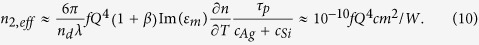


Using 100 nm spacing between nanoparticles leads to 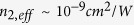 a value more than three orders of magnitude higher than the intrinsic nonlinear index of Si itself.

## Numerical simulation results

**Figure 2:**
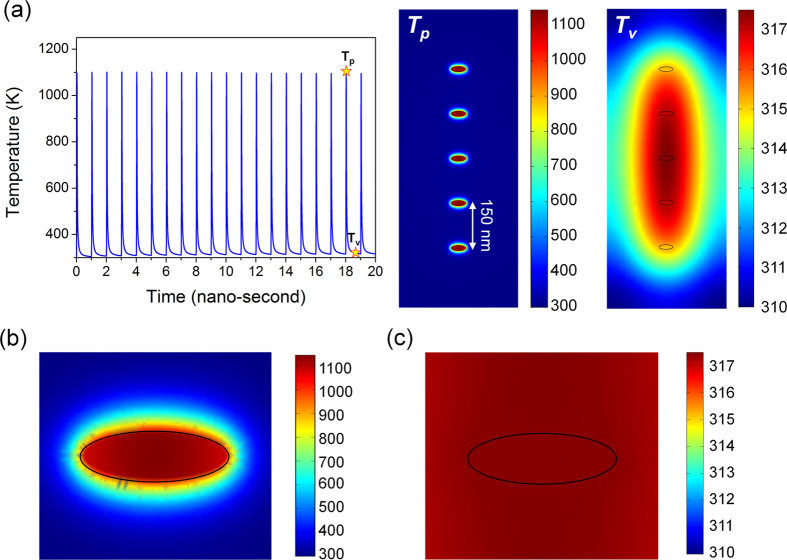
Temperature temporal and spatial profiles in the waveguide under pulsed excitation. (**a**) Temperature pulses at the center of an Ag nanodisk and temperature distribution in the waveguide corresponding to the instant at pulse peak *T*_*p*_ and during the time interval between pulses *T*_*v*_. Magnified views show the same data with finer spatial resolution around an Ag nanodisk in (**b**,**c**) at those same times.

In order to test the results of the order-of-magnitude analytical estimate, we have carried out numerical simulations of the thermal-optical effect in the Ag-nanodisks impregnated Si waveguide (Fig. 1a) by solving three dimensional heat transfer and Maxwell equations using COMSOL Multiphysics. As mentioned above, the dimensions of the Ag nanodisks were chosen such that its SPP resonance occurs at the wavelength of 1550 nm, a value consistent with the calculated optical absorption spectrum of a propagating wave polarized along the long axis of the nanodisk (Fig. 1b). In the case of a single embedded Ag nano-disk, the numerical simulation showed optical absorption of ~23%. Including only five Ag nanodisks in the waveguide would predict complete absorption as shown in Fig. 1a. Figure 1c shows the electric field distribution of the SPP mode in and around an Ag nanodisk. The SPP field is clearly the strongest just outside of the Ag nanodisk near its two tips. The field enhancement is also the highest at 1550 nm and its value has a spatial dependence. Figure 1b also shows the average field enhancement *Q*_*m*_ inside the Ag nanodisk, calculated as the ratio of root-mean-square (RMS) value of the electric field within the Ag nanodisk to that of the incident wave,


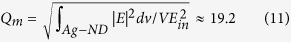


where the electric field squared 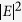 is averaged over the volume of a Ag nanodisk, *V*. Using a value of 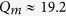 in equation Eq. (4), we obtain 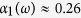, in excellent agreement with our numerical result of 0.23 at the peak of the absorption spectrum in Fig. 2(b).

We next simulate the thermal effect caused by the Ag-nanodisk heat dissipation of a pulsed laser beam propagating along the a-Si waveguide. The time-dependent temperature *T* distribution can be obtained by solving the heat-transfer equation driven by a heat source with the power density *S* as


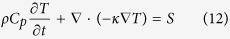


where *ρ* is the mass density equal to 1.035 × 10^4^ *kg*/*m*^3^ for Ag and 2.328 × 10^3^ *kg*/*m*^3^ for a-Si; *C*_*p*_ is the heat capacity at constant pressure, 250 *J*/*kg* · *K* for Ag and 800 *J*/*kg* · *K* for Si; *κ* is the thermal conductivity 430 *W*/*m* · *K* for Ag and 10 *W*/*m* · *K* for a-Si. The bottom surface of silicon waveguide is attached to a perfect heat sink at room temperature 300 K, while other surfaces are exposed to ambient temperature of 300 K. The Ag nanodisks absorbing power from the propagating wave serve as heat sources with power density *S* related to the average field inside of the Ag-nanodisk 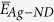 as


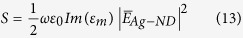


The incident wave under consideration is a laser pulse operating at 1550 nm, with a repetition rate of 1 GHz and a pulse duration of 20 ps. With the peak incident intensity of 1.8 MW/cm^2^, corresponding to 1.5 mW of input power for the waveguide with 220 ×  400-nm^2^ cross section, the peak power density of the heat source is *S* = 5 × 10^20^ *W*/*m*^3^. We allowed a sufficient number of pulses to establish a steady state before performing the detailed thermal analysis, as shown in Fig. 2a. With a thermal diffusion time on the order of a few tens of picoseconds, the temperature rises shortly after the peak of the optical pulse and then relaxes to the average value, only a few Kelvin degrees higher than the the 300 K ambient temperature. The temperature distributions in the waveguide at the peak (*T*_*p*_) of the heat pulse as well as in the interval between pulses (*T*_*v*_) are shown in Fig. 2a, and magnified views of the same data around a single nanoparticle are shown in Fig. 2b,c, respectively.

Following an optical pulse, the high temperatures are concentrated on the metal nanodisks and thin regions of a-Si around them (Fig. 2b), but within 50 ps the temperature is nearly uniformly distributed (Fig. 2c) as heat diffusion dissipates the heat. As mentioned above, the temperature falls down to only a few Kelvins above the 300 K ambient temperature. This is expected as the heat generated inside the small fraction of volume f = 0.064% occupied by the nanodisks spreads over the entire waveguide volume.

**Figure 3:**
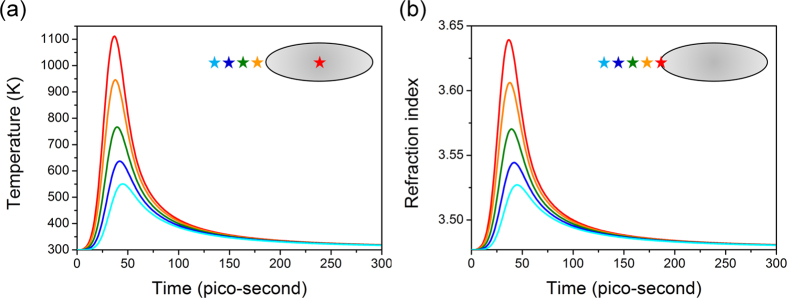
Temporal dependence of temperature. (**a**) Temperature inside the Ag nanodisk (center) and in nearby a-Si (1, 3, 5 and 7 nm away from the nanodisk tip) and (**b**) refractive index in a-Si at 0, 1, 3, 5 and 7 nm away from the nanodisk tip.

In Fig. 3a, a temporal dependence of temperature is shown for several locations, such as at the center of the nanodisk as well as at distances of 1, 3, 5 and 7 nm away from the tip of nanodisk. Using the value of thermo-optical coefficient for a-Si 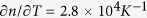
18, we obtain the change of refractive index in a-Si at those same positions as shown in Fig. 3b. The refractive index in a-Si changes from 3.477 in the time interval between pulses to 3.702 at the peak of the pulse – a 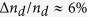 change accomplished in a few tens of picoseconds – a major change of a scale unattainable with any other known type of optical nonlinearity .

**Figure 4:**
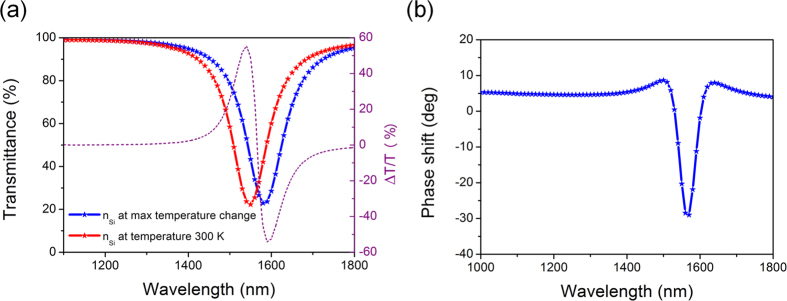
Transmittance and phase shift of the waveguide. (**a**) Transmittance spectra for a-Si waveguide with 5 nanodisks at two temperatures: one at pulse peak (*T*_*p*_) and another at a time in the interval between pulses (*T*_*v*_). The purple dotted curve shows the relative change in transmittance. (**b**) Phase shift as a function of wavelength over the length of the waveguide with five Ag nanodisks.

Now, according to our theory, the change of refractive index around the nanodisk is expected to shift the SPP resonance 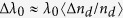 where averaging is over the effective volume. Indeed, as shown by numerical calculations in Fig. 4a the SPP resonance experiences a roughly 50 nm shift with corresponding ~60% relative transmittance change (purple dotted curve). This shift is expected to produce a phase change, and as shown in Fig. 4b (once again obtained by exact numerical modeling), a phase shift of about 40 degrees at the resonance frequency is found, a figure that is in good agreement with our theoretical prediction of 36 degrees made in a previous section. The energy that causes this phase shift is only 30 fJ/bit; full switching requires a 90 degree shift, leading to a prediction of switching energy on the order of 100 fJ/bit, a value consistent with the prediction of 150 fJ/bit made in the previous sections and better than most currently available nonlinear switches.

## Conclusions

We have shown that thermal nonlinearities, commonly believed to be very slow and thus unsuitable for all-optical switching, can become attractively fast when they occur on nanoscale in the plasmonic metamaterials where the optical fields are concentrated in close proximity to the metal nanoparticles. We have shown analytically that using thermally-induced index change of the dielectric material is preferential to using thermal nonlinearity of the metal itself and leads to resonantly-enhanced change of effective index and absorption of the metamaterial. Using example of numerical simulation of short (less than 1 mm long) amorphous Si waveguide impregnated with Ag nanodisks, we have demonstrated that a 60% change of transmission and a phase change of tens of degrees can be optically induced on a ten-picosecond time scale with a peak input optical power of only a milliwatt . While we have made several important assumptions, especially about diffusive rather than ballistic transport, these results provide an order-of magnitude estimate sufficient to stimulate interest in the experimental community for experimental validation of this approach. Using materials with higher thermo-optical coefficients, including phase change materials^25^,^26^,^27^, and optimization of the shape and size of metallic inclusions should further improve the expected performance; experimental results that confirm the thermal nonlinearity approach will lead to low-power, ultrafast, all-optical devices.

## Method

The analysis conducted in this work was based on a combination of analytical derivations and numerical simulations. All simulation results are obtained using the software package COMSOL Multiphysics. The plasmonic waveguide under consideration has a cross section of 220 × 400 nm^2^ consisting of amorphous silicon impregnated with 5 silver nanodisks separated by 150 nm from each other. The incident wave entering one end of the waveguide is a laser pulse operating at 1550 nm with a repetition rate of 1 GHz and a pulse duration of 20 ps. The peak incident intensity is fixed at 1.8 MW/cm^2^, corresponding to 1.584 mW of input power. The permittivity of silver in the spectral region of interest is described by fitting the Drude model with experimental values^28^ for the bulk plasma frequency *ω*_*pl*_ = 9.01 *eV*, and the damping constant *γ* = 0.04 *eV* (two times larger than the experimental bulk value owing to surface scattering and grain boundary effects in the real system). For the thermodynamic simulation, the bottom surface of the a-Si waveguide is pinned at room temperature 300 K, and others are exposed to ambient temperature of 300 K into account. We have used thermal conductivity of a-Si and Ag of 10 and 430 *W*/*m* · *K*, thermal capacity of 800 and 250 *J*/*kg* · *K*, density of 2328 and 10350 *kg*/*m*^3^, respectively.

## Additional Information

**How to cite this article**: Khurgin, J. B. *et al*. Ultrafast Thermal Nonlinearity. *Sci. Rep*. **5**, 17899; doi: 10.1038/srep17899 (2015).
